# High-Fidelity CT Image Denoising with De-TransGAN: A Transformer-Augmented GAN Framework with Attention Mechanisms

**DOI:** 10.3390/bioengineering12121350

**Published:** 2025-12-11

**Authors:** Usama Jameel, Nicola Belcari

**Affiliations:** 1 Department of Computer Science, University of Pisa, 56127 Pisa, Italy; u.jameel@studenti.unipi.it; 2 Department of Physics, University of Pisa, 56127 Pisa, Italy

**Keywords:** CT image, denoising, attention mechanism, image processing, generative adversarial networks, vision transformer

## Abstract

Low-dose computed tomography (LDCT) has become a widely adopted protocol to reduce radiation exposure during clinical imaging. However, dose reduction inevitably amplifies noise and artifacts, compromising image quality and diagnostic confidence. To address this challenge, this study introduces De-TransGAN, a transformer-augmented Generative Adversarial Network specifically designed for high-fidelity LDCT image denoising. Unlike conventional CNN-based denoising models, De-TransGAN combines convolutional layers with transformer blocks to jointly capture local texture details and long-range anatomical dependencies. To further guide the network toward diagnostically critical structures, we embed channel–spatial attention modules based on the Convolutional Block Attention Module (CBAM). On the discriminator side, a hybrid design integrating PatchGAN and vision transformer (ViT) components enhances both fine-grained texture discrimination and global structural consistency. Training stability is achieved using the Wasserstein GAN with Gradient Penalty (WGAN-GP), while a composite objective function—L1 loss, SSIM loss, and VGG perceptual loss—ensures pixel-level fidelity, structural similarity, and perceptual realism. De-TransGAN was trained on the TCIA LDCT and Projection Data dataset and validated on two additional benchmarks: the AAPM Mayo Clinic Low Dose CT Grand Challenge dataset and a private clinical chest LDCT dataset comprising 524 scans (used for qualitative assessment only, as no NDCT ground truth is available). Across these datasets, the proposed method consistently outperformed state-of-the-art CNN- and transformer-based denoising models. On the LDCT and Projection dataset head images, it achieved a PSNR of 44.9217 dB, SSIM of 0.9801, and RMSE of 1.001, while qualitative evaluation on the private dataset confirmed strong generalization with clear noise suppression and preservation of fine anatomical details. These findings establish De-TransGAN as a clinically viable approach for LDCT denoising, enabling radiation reduction without compromising diagnostic quality.

## 1. Introduction

Medical imaging is a cornerstone of modern clinical practice, offering fine-grained, multidimensional insights into the human body. It enables clinicians to detect abnormalities, monitor disease progression, and plan treatments with increasingly greater precision. Imaging modalities such as X-ray, computed tomography (CT), magnetic resonance imaging (MRI), and ultrasound have transformed diagnostic workflows by enabling non-invasive visualization of internal structures [1,2]. Each modality serves distinct clinical purposes: X-ray is frequently used to assess bone fractures and joint integrity [3], MRI provides superior contrast for soft tissues including the brain, spinal cord, and musculoskeletal system [2], and ultrasound supports real-time imaging for cardiac and fetal assessments due to its safety and portability [3]. However, among these, CT imaging stands out due to its ability to generate detailed cross-sectional views, proving especially valuable in detecting tumors, hemorrhages, and organ-level pathologies [1,4]. Despite its diagnostic advantages, CT imaging involves ionizing radiation, which can pose long-term health risks. Studies have shown that excessive exposure to X-rays can cause metabolic disorders, increasing the risk of conditions such as leukemia and cancer [5]. To reduce radiation-related harm, low-dose computed tomography (LDCT) has become a widely adopted protocol in clinical settings. LDCT aims to reduce patient radiation exposure during imaging while preserving diagnostic quality. Traditionally, dose reduction has been achieved by decreasing the tube current and voltage during acquisition [6,7,8]. However, this is only one aspect of modern dose optimization. Contemporary strategies also include the use of automated exposure control (AEC), tube current modulation (TCM), optimized scan parameters (e.g., pitch, rotation time), iterative reconstruction algorithms, and advanced detector technologies. Together, these approaches form a multi-faceted framework for radiation dose minimization, as discussed in [9,10].

Denoising is not only beneficial for enhancing visual quality but also crucial for improving the reliability of downstream tasks such as segmentation, registration, and image fusion, particularly important in computer-aided diagnosis and radiation therapy planning [6]. In CT imaging, denoising also involves the suppression of streak artifacts from metal implants or limited-angle acquisition and clarification of soft-tissue boundaries [1,11]. Similar benefits are observed across other modalities: in MRI, denoising improves the signal-to-noise ratio, in X-ray, it enhances bone visibility, and in ultrasound, it improves fetal and abdominal assessments [2,3]. Consequently, denoising has evolved from a pre-processing step into a clinical necessity that directly impacts diagnostic accuracy and patient outcomes.

In the existing literature, various denoising techniques have been explored to improve image quality in medical imaging, particularly in CT applications where noise severely compromises diagnostic accuracy. Classical approaches such as nonlocal means (NLMs) [12], bilateral filtering [13], and wavelet thresholding [14] have been widely applied across modalities, primarily functioning by averaging or thresholding pixel intensities to suppress noise. While these methods can reduce high-frequency variations, they often introduce oversmoothing, blurring critical anatomical details [15]. Moreover, as handcrafted techniques, they struggle to generalize across diverse clinical scenarios with varying noise distributions and anatomical complexity. To address these shortcomings, earlier algorithms focused on spatial filters such as Gaussian and median filters, followed later by more advanced techniques including nonlocal means (NLMs) and frequency-based approaches (e.g., Fourier and wavelet transforms) [3]. Among these, wavelet-based denoising strikes a balance between multiscale detail preservation and computational efficiency [16]. Nevertheless, even such advanced classical methods frequently suffer from edge blurring and limited adaptability when applied to heterogeneous clinical datasets.

With the advent of deep learning, Convolutional Neural Networks (CNNs) have become the mainstream solution for image denoising, leveraging data-driven learning to adapt to complex noise distributions [5]. Architectures such as U-Net and Denoising Convolutional Neural Network (DnCNN) have demonstrated strong performance in denoising tasks by learning hierarchical representations [5,17]. These CNN-based methods effectively capture local patterns and textures, and have been applied successfully in medical image reconstruction [18]. However, their reliance on fixed-size convolutional kernels limits their receptive field, restricting the model’s ability to extract global contextual information. In LDCT imaging, where subtle long-range structures are critical (e.g., vessels, tissue boundaries), this limitation becomes a bottleneck.

**Figure 1:**
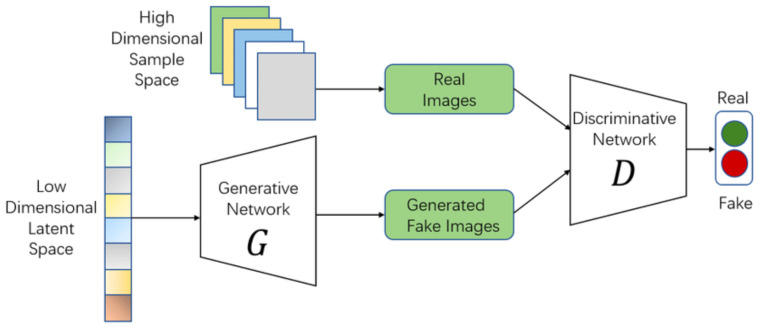
Baseline GAN architecture used in this study for LDCT denoising.

To address this issue, Generative Adversarial Networks (GANs) have been increasingly adopted for medical image restoration, particularly in LDCT denoising [7,19,20]. GANs consist of two components: a generator that synthesizes denoised images and a discriminator that attempts to distinguish between real and generated images. Through adversarial training, the generator learns to produce outputs that are perceptually and statistically similar to ground truth images. Unlike traditional pixel-wise loss functions, GANs are capable of recovering fine anatomical textures and preserving perceptual realism [8,20,21]. Their potential has been recognized across many CT denoising pipelines. Figure 1 illustrates the baseline GAN architecture typically employed for this task.

While GAN-based methods provide visually appealing results, recent advances have shown that their performance can be further enhanced by integrating attention mechanisms. Originally developed for natural language processing [17,22,23], attention models allow networks to focus dynamically on the most informative regions of an input. These mechanisms have since been adapted for various vision tasks such as segmentation, classification, and image restoration. In LDCT imaging, attention modules are especially useful for suppressing background noise while focusing on clinically significant regions like soft-tissue boundaries, lesions, or vessel structures [24,25]. They offer a way to guide the model’s learning process more precisely toward diagnostic relevance. Motivated by these insights, we propose denoising transformer-augmented Generative Adversarial Network (De-TransGAN), a novel attention-guided denoising framework built upon the transformer-augmented GAN architecture. The generator in De-TransGAN fuses convolutional layers with transformer blocks to extract both local and long-range features, addressing CNN’s limitation in global modeling. To further enhance feature representation, we embed channel and spatial attention modules derived from the Convolutional Block Attention Module (CBAM) [26]. These modules help the model emphasize regions that are more vulnerable to noise and critical for diagnosis. On the discriminator side, we design a hybrid discriminator combining the PatchGAN architecture with a vision transformer (ViT) [27], improving the ability to assess both textural quality and global consistency in the generated images. Training is stabilized using the Wasserstein GAN with Gradient Penalty (WGANGP) [19], which mitigates common GAN training issues like mode collapse and convergence instability.

Our model is further optimized through a composite loss function comprising L1 loss, Structural Similarity Index (SSIM) loss, and VGG perceptual loss. This multi-objective loss formulation ensures accurate pixel-level reconstruction, structure preservation, and perceptual fidelity, respectively. By integrating these components, De-TransGAN is designed to deliver denoised CT images that not only minimize noise but also retain subtle anatomical features necessary for reliable diagnosis. The central motivation behind De-TransGAN is to enable high-fidelity LDCT image reconstruction without the need to revert to higher radiation doses. As explored in the following sections, many existing denoising models fail to preserve detail in low-contrast or artifact-prone regions. Our proposed approach aims to overcome these gaps by harnessing the strengths of transformer-based modeling, attention-guided refinement, and adversarial learning [28]. To validate its effectiveness, we conducted experiments on three diverse datasets, demonstrating both strong quantitative results and generalization across data sources. Therefore, the main contributions of this work are summarized as follows:

- We design a transformer-based generator enhanced with CBAM-based attention to jointly model long-range dependencies and focus on diagnostically relevant features.
- We introduce a composite loss function (L1 + SSIM + VGG) to balance pixel-level accuracy, structural similarity, and perceptual quality during training.
- We propose a hybrid discriminator combining PatchGAN and vision transformer (ViT) to improve the network’s ability to evaluate textural realism and global coherence.
- We stabilize adversarial training through the use of the Wasserstein GAN with Gradient Penalty (WGAN-GP), ensuring robust convergence and image quality.
- We perform extensive evaluations on three datasets—the LDCT and Projection dataset (used for training and testing), the AAPM Mayo Clinic dataset, and a private clinical LDCT dataset—demonstrating that De-TransGAN consistently outperforms baseline and state-of-the-art methods.

## 2. Related Work

Given the increasing importance of LDCT imaging in clinical diagnostics, numerous denoising algorithms have been developed to reduce radiation-induced noise while preserving diagnostic quality. This section reviews recent advancements in LDCT denoising, with emphasis on transformer-augmented architectures, GAN-based frameworks, attention-guided designs, and lightweight solutions. Each study is analyzed in terms of its technical contribution, architectural novelty, evaluation dataset, and inherent limitations, building the rationale for our proposed model.

Yan et al. [29] proposed the Channel Graph Perception Uformer (CGP-Uformer), a Uformer variant that incorporates Channel Graph Perception (CGP) modules to enhance interchannel feature learning. Trained on the AAPM Mayo Clinic 2016 dataset with 5000 paired low-dose CT (LDCT)/full-dose CT (FDCT) images, the model achieved an average PSNR of 35.56 ± 3.69 dB and SSIM of 0.9221. While outperforming traditional CNN-based architectures, its substantial memory footprint and computational complexity restrict scalability for real-time clinical deployment [30]. Similarly, a multi-attention U-Net was introduced in [22,31,32], combining local, channel, and hierarchical attention mechanisms to improve feature extraction under noisy conditions. Tested on QIN-LUNG-CT and Mayo datasets, it reached PSNRs of 34.73 and 28.91 dB with SSIMs of 0.93 and 0.86, respectively. However, the absence of global attention mechanisms limited its ability to capture long-range anatomical dependencies, restricting perceptual fidelity.

To address structure blurring, Compound Feature Attention Network (CFAN-Net) [23] integrated compound feature attention with edge-enhancement modules. Although achieving strong results (PSNR: 33.97 ± 0.40 dB, SSIM: 0.9198) on a small set of 35 Mayo images, its limited sample size raised concerns about generalization and clinical reproducibility. Moreover, its real-time feasibility was not assessed. In parallel, Wang et al. [28] introduced Hierarchical Transformer (Hformer), a vision transformer-based model with residual learning and scale encoding for global context modeling. Despite its innovative design, the lack of quantitative benchmarks and detailed implementation hindered reproducibility and adoption.

The sinogram-domain Image Synthesis Transformer model (SIST) [33] pursued a unique strategy by denoising in the sinogram domain prior to image reconstruction. It achieved up to 44.74 dB PSNR and 0.916 SSIM. While demonstrating strong results, reliance on raw sinogram data limits its clinical utility, as sinograms are not routinely available in hospital workflows. Zhang et al. [34] explored sparse-view artifact removal using a transformer-augmented GAN with multidimensional attention. Although showing qualitative improvements over previous transformer-based and GAN-based baselines, quantitative benchmarking and validation on standard LDCT datasets were missing, limiting its broader relevance [35]. Edge-enhancement transformer (Eformer) [36] tackled edge blurring by fusing transformer blocks with Sobel–Feldman edge filters, reporting PSNR 43.487 dB and SSIM 0.9861 on the Mayo dataset.

Chen et al. [37] further synthesized the attention-based GAN literature, particularly in segmentation and denoising, providing valuable perspectives but lacking experimental validation or direct benchmarking. Recent advances in medical imaging have leveraged attention-guided and transformer-based models for improved segmentation, diagnosis, and reconstruction. Notable examples include AI-generated annotations for segmentation, attention-guided CNNs for early diabetic retinopathy detection, and cross-scale transformer methods for super-resolution [38,39].

Wang et al. [18] proposed CTformer, a convolution-free Token2Token Vision Transformer designed for LDCT denoising, which surpassed baselines such as Residual Encoder–Decoder Convolutional Neural Network (RED-CNN) and DnCNN on the Mayo dataset. Although promising, CTformer’s performance was highly sensitive to tokenization strategies, highlighting design fragility. Patwari et al. [40] attempted to mitigate parameter inefficiency by introducing a lightweight reinforcement learning framework with bilateral filtering. While offering modest improvements (PSNR↑ 0.4 dB, SSIM↑ 0.025), its tuning complexity and scalability concerns reduced practical viability. In [41], a post-processing GAN pipeline refined LDCT reconstructions with qualitative success, but absence of numerical metrics limited rigorous comparison. Similarly, You et al. [42] demonstrated a veterinary-specific GAN with anti-aliasing and multiscale discrimination, which enhanced canine LDCT clarity but lacked relevance for human clinical imaging.

In summary, Table 1 provides a critical analysis of recent LDCT denoising methods. A cross-comparison reveals persisting challenges: (1) transformer-heavy models with excessive memory and computation requirements that preclude real-time deployment [29]; (2) CNN-based denoisers constrained by insufficient global context modeling [22]; (3) over-reliance on small datasets (e.g., 35 Mayo images), undermining generalization and reproducibility [23]; (4) loss functions dominated by single metrics (L1 or SSIM), failing to preserve perceptual realism [22,23]; and (5) underexplored hybrid architectures where transformers, GANs, and attention are not fully leveraged in synergy. Motivated by these observations, our proposed De-TransGAN integrates transformer blocks, CBAM-guided attention, a hybrid PatchGAN-ViT discriminator, and a composite loss under the WGAN-GP framework. This design directly addresses the computational, structural, and perceptual gaps identified, enabling robust and clinically meaningful LDCT denoising.

## 3. Method

### 3.1. Background

**Figure 2:**
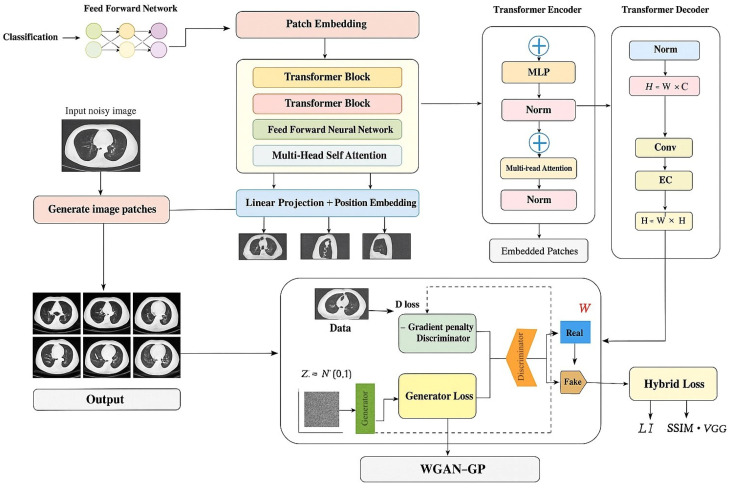
Architecture of the proposed De-TransGAN: Attention-Guided Transformer–GAN framework for LDCT image denoising.

Low-dose CT (LDCT) denoising presents unique challenges where traditional approaches including spatial filtering, frequency transforms, and CNN-based models often fail to jointly preserve fine anatomical details and capture the global context required for diagnostic fidelity. Classical filters tend to oversmooth subtle structures, while CNNs are inherently limited by their local receptive fields, making them less effective in modeling long-range dependencies critical for tissues, vessels, and lesion boundaries. GAN-based frameworks have improved perceptual quality but remain prone to unstable training and difficulty in balancing realism with structural accuracy. To overcome these limitations, we propose De-TransGAN, an attention-guided transformer-augmented GAN framework, illustrated in Figure 2. The proposed architecture integrates transformer blocks, CBAM-guided attention, a hybrid PatchGAN-ViT discriminator, and a composite loss under the WGAN-GP formulation. This unified design directly addresses the computational, structural, and perceptual gaps identified in prior studies, enabling robust and clinically meaningful LDCT denoising. The framework begins with a transformer-based generator that integrates convolutional layers and self-attention blocks to simultaneously extract local features and model long-range dependencies. Channel and spatial attention modules further refine feature learning by guiding the network toward noise-prone and diagnostically relevant regions. The discriminator is upgraded under the WGAN-GP formulation, combining adversarial learning stability with the capacity to distinguish fine textural variations between real full-dose CT images and generated outputs. Training is driven by a hybrid loss function comprising pixel-wise L1 loss, Structural Similarity Index (SSIM) loss, and VGG-based perceptual loss, ensuring fidelity across intensity accuracy, structural preservation, and perceptual realism. Gradients from these complementary objectives are iteratively propagated through the network, progressively refining the generator and discriminator to deliver reconstructions of high diagnostic quality. In contrast to conventional GAN-based denoising pipelines, De-TransGAN introduces multiple tailored enhancements, namely attention-guided transformer modeling, hybrid discrimination, stabilized adversarial learning, and composite losses, that collectively enable robust LDCT denoising. The following subsections detail each of these components.

### 3.2. Transformer-Based Generator

Conventional GAN generators designed with CNN backbones are inherently limited in modeling long-range dependencies due to their fixed-size convolutional kernels. While they effectively capture local textural patterns, their inability to encode broader anatomical context restricts their performance in LDCT denoising, where global structural awareness is essential for preserving vessels, organ boundaries, and subtle lesions. To address these limitations, we replace the baseline CNN generator with a customized transformer-based architecture inspired by TransGAN [43].

In the proposed design, the generator employs vision transformer (ViT) blocks that divide the input image into a set of non-overlapping patches, as illustrated in the ‘Patch Embedding’ stage of Figure 2, and model inter-patch relationships through self-attention. This patch-based representation enables the network to move beyond local filtering, capturing global contextual cues that are critical for distinguishing noise from subtle diagnostic features. The generator’s transformer blocks follow a ViT-inspired architecture with 16 × 16 patch embedding, 768-dimensional tokens, 6 encoder–decoder layers, 8-head self-attention, and MLP layers of width 2048, incorporating residual connections and positional encoding. To further enhance representation power, multi-head self-attention (MHSA) layers are embedded between residual transformer blocks, allowing the model to attend to spatially distant yet semantically related regions. This mechanism provides a natural framework for suppressing noise patterns consistently across large anatomical areas while preserving clinically relevant fine structures such as vessel walls or micro-lesions.

Operationally, the generator accepts a noisy CT slice x and processes it through an encoder–decoder hierarchy of transformer layers with skip connections to retain both global and local information. Formally, the procedure begins by splitting x into non-overlapping patches $\left\{p_{1},\ldots ,p_{n}\right\}$, each embedded via a function E(·) with added positional encodings. These embeddings are passed through *L* stacked transformer blocks, each comprising MHSA, a feed-forward multilayer perceptron (MLP), and residual connections. The MHSA mechanism can be expressed as

(1) $$\mathrm{MHSA}\left(Q,K,V\right)=\mathrm{Concat}\left(head_{1},\ldots ,head_{h}\right)W^{o},$$

where each attention head is defined as

(2) $$head_{i}=\mathrm{Softmax}\left(\frac{QW_{i}^{Q}{\left(KW_{i}^{K}\right)}^{T}}{\sqrt{d_{k}}}\right)VW_{i}^{V}.$$

The final representation $z_{L}$ is then decoded through the symmetric transformer decoder to reconstruct the denoised output $\hat{x}$, which retains both fine anatomical detail and global consistency. This transformer-augmented generator forms the core of the proposed De-TransGAN framework.

### 3.3. Attention Mechanisms

While transformer blocks provide global context modeling, their effectiveness in medical image denoising can be further enhanced by explicitly guiding the network toward diagnostically relevant regions. To this end, we integrate channel and spatial attention modules, inspired by the Convolutional Block Attention Module (CBAM), directly within the transformer layers of the generator. CBAMs are inserted after selected transformer blocks in both the encoder and decoder, sequentially applying channel attention (MLP over pooled features) followed by 7 × 7 spatial attention convolution, as described in [26]. These modules refine intermediate feature maps, allowing the network to emphasize critical structures while suppressing irrelevant noise.

The *channel attention* mechanism explores the relative importance of each feature map, enabling the model to selectively amplify channels that contribute most to structural detail (e.g., vessel edges, tissue boundaries). It is defined as

(3) $$M_{c}\left(F\right)=\sigma \left(\mathrm{MLP}(\mathrm{AvgPool}(F))+\mathrm{MLP}(\mathrm{MaxPool}(F))\right)$$

where *F* is the input feature map of shape (C,H,W), $M_{c}$ is the channel attention response of size (C,1,1), and σ denotes the sigmoid activation. Global average pooling and max pooling summarize spatial information, followed by a shared MLP that generates the attention weights.

Complementing this, the *spatial attention* mechanism enhances local structure preservation by highlighting informative regions within each slice while suppressing background noise. It is computed as

(4) $$M_{s}\left(F\right)=\sigma \left(f^{7\times 7}\left([\mathrm{AvgPool}(F);\mathrm{MaxPool}(F)]\right)\right)$$

where $f^{7\times 7}$ represents a convolution with a 7×7 kernel applied to the concatenated average- and max-pooled maps.

The final refined feature representation is obtained as

(5) $$F^{\prime }=M_{c}\left(F\right)\cdot F,\;F^{\prime \prime }=M_{s}\left(F^{\prime }\right)\cdot F^{\prime }$$

where $F^{\prime \prime }$ serves as the input to subsequent transformer layers. This sequential refinement ensures that the generator maintains both global contextual awareness and localized diagnostic detail, enabling more accurate noise suppression in LDCT reconstructions.

### 3.4. Hybrid Discriminator Design

In adversarial learning for medical image denoising, the discriminator plays a pivotal role in assessing the realism of the generated images. By providing feedback to the generator, it ensures that the denoised CT reconstructions are not only pixel-wise accurate but also perceptually plausible, capturing both subtle anatomical details and overall structural consistency. In the final implementation, the ViT-based discriminator was adopted for reporting all quantitative and qualitative results, as it achieved higher perceptual fidelity and global consistency compared to PatchGAN.

#### 3.4.1. PatchGAN Discriminator

The PatchGAN discriminator operates on local overlapping patches rather than the entire image, classifying each patch as real or fake. This design enforces the generator to produce fine-grained realism, ensuring that high-frequency textures such as vessel edges, tissue boundaries, and micro-lesions are preserved. PatchGAN has been widely adopted in image restoration tasks where maintaining local anatomical fidelity is critical.

#### 3.4.2. ViT-Based Discriminator

Complementing PatchGAN, we also use a vision transformer (ViT)-based discriminator that models long-range dependencies across the entire image. Leveraging multi-head self-attention, this discriminator evaluates global coherence, enabling the detection of structural inconsistencies that may not be evident at the patch level. By capturing inter-patch relationships, the ViT discriminator strengthens the assessment of overall image quality and clinical plausibility.

#### 3.4.3. Hybrid Evaluation

In practice, both discriminators are trained and compared to analyze trade-offs between local realism and global consistency. While PatchGAN enforces anatomical texture preservation, the ViT-based discriminator enhances holistic assessment. Together they provide complementary supervision signals, driving the generator toward reconstructions that are simultaneously locally detailed and globally coherent. In our experimental setup, the ViT-based discriminator has no impact on real-time deployment or inference latency, as only the generator is used during testing while the discriminator remains inactive at inference time.

### 3.5. WGAN-GP for Stable Adversarial Training

Conventional GAN frameworks often suffer from training instabilities such as vanishing gradients, mode collapse, or oscillatory convergence, which can severely degrade the fidelity of reconstructed medical images. In LDCT denoising, these issues manifest as loss of anatomical structures, unrealistic textures, or inconsistent reconstructions, all of which undermine clinical reliability. To mitigate these challenges, we adopted the Wasserstein GAN with Gradient Penalty (WGAN-GP) framework [19]. The Wasserstein distance provides a smooth and continuous metric to measure the divergence between the real and generated data distributions, allowing the discriminator (critic) to provide more informative gradients. Meanwhile, the gradient penalty enforces the Lipschitz constraint directly, avoiding the limitations of weight clipping and ensuring stable optimization. The discriminator loss under WGAN-GP is formulated as

(6) $$L_{D}=E\left[D\left(\hat{x}\right)\right]-E\left[D\left(x_{gt}\right)\right]+\lambda_{gp}\;E_{\tilde{x}}\left[{\left({∥\nabla_{\tilde{x}}D\left(\tilde{x}\right)∥}_{2}-1\right)}^{2}\right]$$

where *x* denotes a real full-dose CT image, $\hat{x}$ the generated denoised image, and $\tilde{x}$ a random interpolation between the two. The coefficient $\lambda_{gp}$ controls the strength of the gradient penalty. By adopting WGAN-GP, our adversarial training achieves robust convergence, reduces the risk of mode collapse, and drives the generator to produce denoised CT images with both high perceptual quality and structural fidelity.

### 3.6. Hybrid Loss Function: L1 + SSIM + VGG

The choice of loss function plays a critical role in training denoising networks, as it defines the balance between numerical fidelity, structural preservation, and perceptual realism. In medical image reconstruction, relying on a single loss often leads to suboptimal outcomes: pixel-wise losses preserve intensity accuracy but miss structural similarity, while perceptual losses may produce visually realistic textures at the cost of anatomical fidelity. As demonstrated in prior studies, hybrid loss functions effectively balance pixel-level accuracy, structural fidelity, and perceptual realism [20,44,45]. Therefore, our framework employs a combined loss function consisting of L1, SSIM, and VGG perceptual losses. The effectiveness of this formulation is validated through consistently stable training and superior quantitative results across all evaluated datasets. This multi-objective design ensures accurate pixel reconstruction, enhanced structural consistency, and realistic visual quality.

#### 3.6.1. L1 Loss (Pixel Fidelity)

Also referred to as the Mean Absolute Error (MAE), L1 loss enforces pixel-wise accuracy by penalizing the absolute differences between predicted and reference images. It ensures that the overall intensity distribution of the denoised image remains close to the ground truth. Mathematically,

(7) $$L_{L1}=\frac{1}{N}\sum_{i=1}^{N}\left|{\hat{x}}_{i}-x_{gt,i}\right|$$

where *N* is the number of pixels, ${\hat{I}}_{i}$ represents the predicted intensity, and $I_{i}$ denotes the ground truth full-dose intensity. While robust to outliers, L1 alone cannot guarantee perceptual sharpness or structural integrity.

#### 3.6.2. SSIM Loss (Structural Preservation)

The Structural Similarity Index Measure (SSIM) loss evaluates image quality based on structural information rather than raw pixel differences. By comparing luminance, contrast, and structure between the predicted and reference images, SSIM captures human visual perception of quality. In LDCT denoising, this component is particularly important for maintaining soft-tissue boundaries and fine anatomical details that pixel-wise losses may blur. The SSIM loss can be expressed as

(8) $$L_{SSIM}=1-SSIM\left(\hat{x},x_{gt}\right)$$

where $\hat{I}$ is the predicted image and *I* is the reference clean image. By minimizing $L_{SSIM}$, the network is encouraged to produce denoised reconstructions that remain structurally consistent with clinical ground truth.

#### 3.6.3. VGG Perceptual Loss

This is used to measure the difference in high-level feature representations extracted from a pretrained VGG-19 network. This encourages visually plausible textures and natural appearance in the output [30]. Therefore, based on these three basic loss functions, the overall generator loss is formulated as a weighted hybrid function (Equation (9)), integrating pixel fidelity, structural integrity, and perceptual quality.

(9) $$L_{\mathrm{total}}=\lambda_{1}L_{L1}+\lambda_{2}L_{SSIM}+\lambda_{3}L_{VGG}$$

where $\lambda_{1}$, $\lambda_{2}$, and $\lambda_{3}$ are empirically chosen weights through grid search over a validation subset of the LDCT dataset.

The complete training and inference procedure of the proposed De-TransGAN framework is formally described in Algorithm 1. This algorithm integrates the generator, discriminator, hybrid loss, and WGAN-GP optimization into a unified workflow, ensuring stable adversarial training and clinically faithful LDCT denoising.
**Algorithm 1:** De-TransGAN: Supervised Training and Inference.
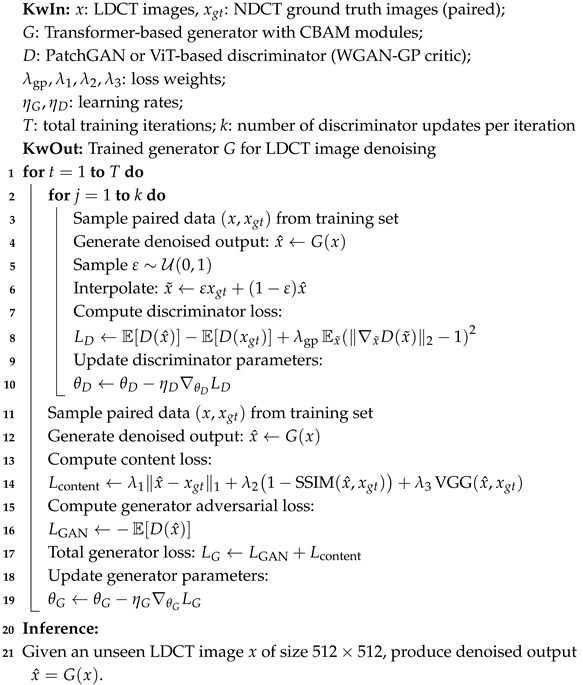


## 4. Dataset

This study relies on three datasets: the LDCT and Projection Data collection [46], the Mayo dataset [47], and an additional private clinical dataset. The following subsections summarize the key characteristics and usage of each dataset in this work.

### 4.1. LDCT and Projection Data

**Figure 3:**
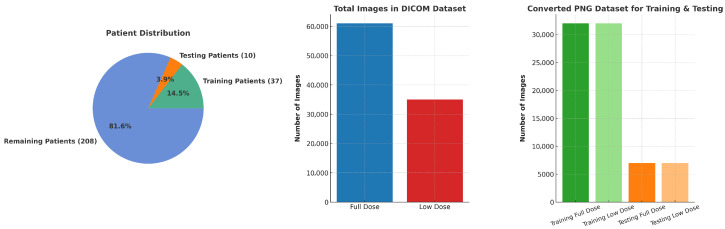
Overview of the LDCT and Projection Data collection, including patient distribution, total DICOM images, and converted PNG subsets for training and testing.

The Low Dose CT Image and Projection Data (LDCT and Projection Data) collection, hosted by The Cancer Imaging Archive (TCIA), is a large-scale resource developed by the Mayo Clinic with support from the National Institute of Biomedical Imaging and Bioengineering [46]. It is specifically designed to advance research in CT image reconstruction, denoising, and radiation dose reduction. The dataset provides paired low-dose CT (LDCT) and full-dose CT (FDCT) images, enabling direct supervision for training and testing denoising models. For this study, the original DICOM files were converted into .png format to ensure compatibility with the proposed framework. We adopted a pre-processing strategy during DICOM to PNG conversion which is consistent with prior work [8] and confirmed that this rescaling preserved all clinically visible features in our approach. Specifically, each DICOM image was normalized independently using clinically appropriate window width and level values, with pixel intensities linearly mapped to 8-bit range based on the maximum value of each individual image. All images were resized to 512×512 pixels for uniformity. We randomly selected 37 patients for training and 10 patients for evaluation, comprising 31,513 and 5365 paired 2D low-dose and full-dose CT images, respectively. This distribution ensured a diverse and balanced representation of anatomical regions and noise levels. An overview of the patient split, total DICOM counts, and PNG subsets is shown in Figure 3.

### 4.2. Mayo Dataset

The Mayo dataset was released as part of the American Association of Physicists in Medicine (AAPM) Low Dose CT Grand Challenge [47]. It contains paired normal-dose and simulated low-dose CT scans from 10 patients. Each case includes reconstructions performed with filtered back projection (FBP) and iterative reconstruction methods at multiple dose levels (e.g., full dose and quarter-dose). The Mayo dataset provides realistic low-dose noise patterns and has become a widely adopted benchmark for evaluating and comparing LDCT denoising approaches. In this study, it was used exclusively for independent validation to assess the comparative performance of our model against established baselines.

### 4.3. Private Dataset

To further assess the generalization capability of the proposed model in real-world clinical practice, we used a private dataset collected from a local hospital under appropriate ethical approval and anonymization protocols. This dataset was provided through a research collaboration agreement with the hospital and was made available strictly for experimental evaluation and testing purposes only. Due to confidentiality restrictions, the dataset cannot be publicly shared. It includes four patients, labeled A001, A002, A003, and A004, containing 100, 161, 158, and 105 images, respectively. All scans were acquired as low-dose CT images and provided in 512×512 resolution. Since corresponding full-dose references were not available, this dataset was used exclusively for qualitative validation. The results on this private dataset demonstrate that the proposed De-TransGAN can generalize effectively to unseen clinical data, producing denoised reconstructions that preserve anatomical fidelity while suppressing noise artifacts.

## 5. Performance Metrics

To objectively evaluate the effectiveness of the proposed LDCT denoising framework, we employed two widely recognized quantitative metrics: Peak Signal-to-Noise Ratio (PSNR) and Structural Similarity Index Measure (SSIM). These metrics are standard in medical image reconstruction studies and provide complementary insights into denoising performance. PSNR measures pixel-level fidelity by quantifying the reconstruction error with respect to reference full-dose images, while SSIM evaluates perceptual quality by capturing structural similarity, contrast, and luminance consistency. Together, PSNR and SSIM offer a comprehensive assessment, balancing numerical accuracy with clinical interpretability, both of which are critical for validating denoised CT images in diagnostic practice.

### 5.1. Peak Signal-to-Noise Ratio (PSNR)

PSNR is a widely adopted objective metric to measure the fidelity of a reconstructed or denoised image relative to a reference (ground truth) image. It is derived from the Mean Squared Error (MSE) between the two images and expresses the logarithmic ratio between the maximum possible signal intensity and the noise power. First, the MSE is computed between the reference image *I* and the reconstructed image *K* as

(10) $$\mathrm{MSE}=\frac{1}{m\times n}\sum_{i=1}^{m}\sum_{j=1}^{n}{\left[\hat{x}\left(i,j\right)-x_{gt}\left(i,j\right)\right]}^{2}$$

Then, PSNR is calculated as

(11) $$\mathrm{PSNR}=10\times \log_{10}\left(\frac{{\mathrm{Max}}^{2}}{\mathrm{MSE}}\right)$$

where Max is the maximum possible pixel value (e.g., 255 for 8-bit grayscale images), and m×n denotes the image size. A higher PSNR indicates better reconstruction quality, with values above 30 dB typically considered acceptable in medical imaging applications.

### 5.2. Structural Similarity Index (SSIM)

SSIM is a perceptual quality metric that evaluates image degradation by considering structural information, luminance, and contrast [42]. Unlike PSNR, which is based on absolute pixel error, SSIM models human visual perception by comparing local patterns of pixel intensities. The SSIM index between two images *x* and *y* is defined as

(12) $$\mathrm{SSIM}\left(x,y\right)=\frac{\left(2\mu_{x}\mu_{y}+C_{1}\right)\left(2\sigma_{xy}+C_{2}\right)}{\left(\mu_{x}^{2}+\mu_{y}^{2}+C_{1}\right)\left(\sigma_{x}^{2}+\sigma_{y}^{2}+C_{2}\right)}$$

where $\mu_{x},\mu_{y}$ are the mean intensities, $\sigma_{x}^{2},\sigma_{y}^{2}$ are the variances, and $\sigma_{xy}$ is the covariance of *x* and *y*. The constants $C_{1}={\left(K_{1}L\right)}^{2}$ and $C_{2}={\left(K_{2}L\right)}^{2}$ stabilize the division, with $K_{1}=0.01$, $K_{2}=0.03$, and L=255 for 8-bit images. SSIM ranges from −1 to 1, where 1 represents perfect structural similarity. In medical image restoration tasks, SSIM values above 0.9 are generally considered indicative of high-quality reconstructions.

### 5.3. Development Environment

All experiments were conducted in PyCharm (v2025.1) using Python 3.9, PyTorch (v2.x), and CUDA 11.8, with additional support from NumPy, OpenCV, scikit-image, and MONAI for medical image analysis. The proposed De-TransGAN framework was trained and evaluated on a high-performance workstation equipped with four NVIDIA A100 GPUs (40 GB memory each), enabling efficient parallelization and accelerated deep learning computation. Preliminary dataset pre-processing and conversion tasks were carried out on a single GPU, while full-scale model training was distributed across all four GPUs to ensure scalability and stability. The end-to-end pipeline, including training, validation, and final testing, required approximately 17 hours to complete, encompassing all optimization and evaluation steps.

## 6. Approaches for Comparative Analysis

To evaluate the performance of the proposed framework against existing methods, the following related approaches were selected:

Cycle-Consistent Generative Adversarial Network (CycleGAN) [48]: An unpaired image-to-image translation model that employs adversarial and cycle-consistency losses to learn mappings between LDCT and FDCT domains without paired data. It eliminates the need for paired LDCT–FDCT datasets, enabling unsupervised denoising by leveraging unpaired datasets and thus increasing applicability in real-world scenarios.

RED-CNN [49]: A supervised residual encoder–decoder network trained on paired LDCT and FDCT images to learn noise residuals and restore high-quality outputs. It addresses the limitations of traditional filters by learning complex noise patterns directly from data and is considered one of the first effective CNN-based denoising models, setting an early benchmark in structure-preserving performance.

Noise2Sim [27]: A self-supervised denoising approach that predicts each pixel using its surrounding noisy context under the assumption of statistical independence of noise. It enables LDCT denoising without clean FDCT labels by relying on internal image redundancy, eliminating dependence on supervised training with ground truth images.

CTformer [18]: A transformer-based architecture designed to capture long-range spatial and temporal dependencies across CT slices via attention mechanisms. It introduced transformers into LDCT denoising, improving structural preservation and contextual consistency in sequential CT data, and addressed CNNs’ limitation in modeling long-range dependencies.

U-Net with Attention and Dense Connections (UNAD) [50]: An unsupervised domain adaptation model that employs adversarial training to denoise LDCT images without paired data or labels. It enhances robustness to domain shift by aligning feature distributions between noisy and clean domains, tackling the generalization issue where models trained on one domain fail on unseen test distributions.

Contextual Contrast Detail Attention Feature Fusion Network (CDAF-Net) [51]: A deep denoising architecture that incorporates a Contextual Contrast Detail Attention (CCDA) module to capture both high-level semantics and fine structural details. It further integrates a Selective Kernel Feature Fusion (SKFF) module to merge shallow encoder and deep decoder features across multiple resolutions. Evaluated on the Mayo LDCT dataset, it achieved state-of-the-art results by effectively balancing global and local feature learning.

## 7. Experiments and Results

In this section, we evaluate the performance of the proposed De-TransGAN by conducting comprehensive comparisons with a range of recent state-of-the-art deep learning techniques for LDCT image denoising. The evaluation covers multiple datasets to assess quantitative performance, visual quality, and generalization across different clinical scenarios.

### 7.1. Performance on LDCT and Projection Dataset

To comprehensively evaluate the proposed framework, we trained and tested De-TransGAN on the LDCT and Projection dataset under varying noise conditions. Importantly, none of the competing methods were originally trained on this dataset, ensuring a fair and unbiased comparison of their generalization ability. A detailed performance analysis on chest CT images from this dataset is presented below.

In terms of computational efficiency, the proposed De-TransGAN achieved an average inference time of 0.037 s per 512 × 512 CT slice and approximately 60 s for a complete CT volume when evaluated on dual NVIDIA A100 GPUs. This efficiency demonstrates the framework’s suitability for near-real-time clinical deployment.

#### 7.1.1. Chest CT Results

**Figure 4:**
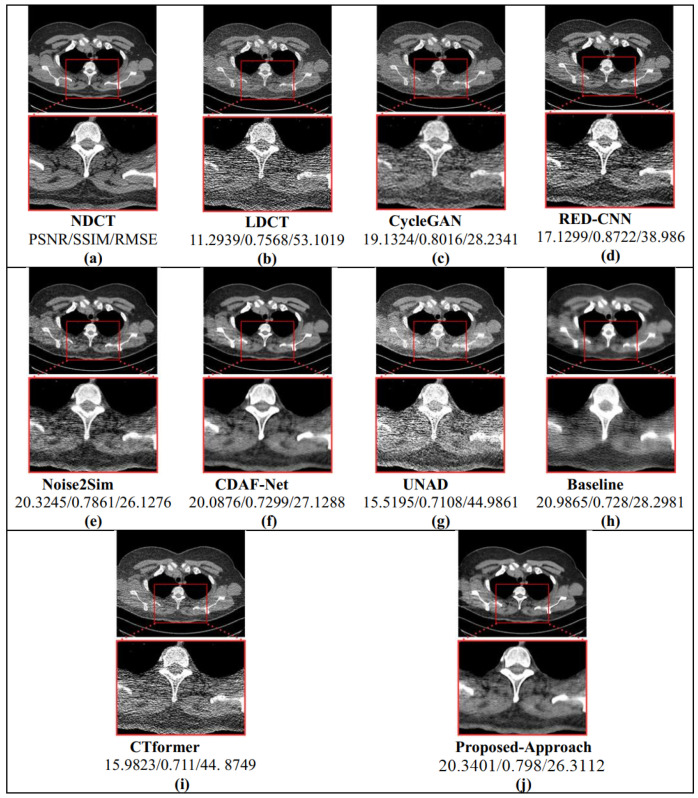
Results showing visualization of low-dose chest CT (LDCT) images, full-dose CT (FDCT) images, and denoised CT images (the top part of each image depicts the complete chest CT scan, while the bottom part of the image shows the enlarged ROI images for each chest CT scan).

**Figure 5:**
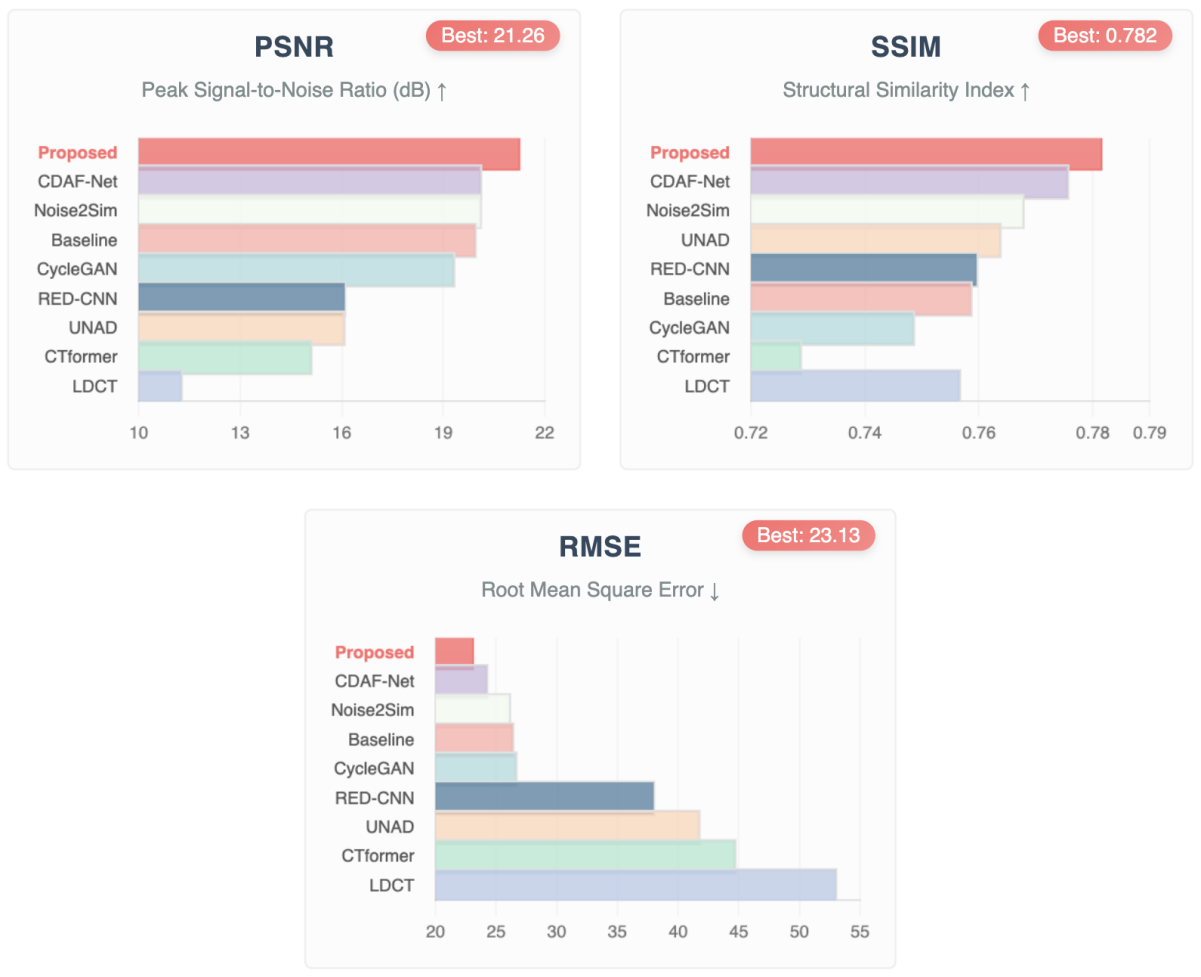
Comparative bar plots of PSNR, SSIM, and RMSE for chest CT images from the LDCT and Projection dataset. The proposed De-TransGAN achieves the highest PSNR (21.26 dB) and SSIM (0.782), along with the lowest RMSE (23.13), demonstrating superior balance between noise suppression and structural fidelity compared to state-of-the-art approaches. *Note: ↑ indicates higher values represent better performance; ↓ indicates lower values represent better performance*.

Chest CT images from the LDCT and Projection dataset were used to evaluate the denoising capability of the proposed framework. As summarized in Table 2 and as shown in Figure 4, and further illustrated in the graphical comparison in Figure 5, the proposed method consistently achieved the best overall performance.

Specifically, De-TransGAN obtained a PSNR of 21.26 dB, SSIM of 0.7816, and the lowest RMSE of 23.13. These results indicate superior fidelity in both structural preservation and noise suppression, with the reduced RMSE reflecting the model’s ability to recover fine anatomical details such as vessels and small lesions that are often blurred in competing approaches. Among the comparative methods, CDAF-Net [51] and Noise2Sim [27] emerged as the closest competitors, achieving PSNRs around 20.1 dB and SSIM values close to the proposed approach. However, their higher RMSE values suggest the persistence of residual noise and less stable reconstructions. Baseline [47] provided moderate results but was consistently outperformed across all three metrics. CycleGAN [48] demonstrated some improvement through cycle-consistency learning but failed to match the structural fidelity of De-TransGAN. RED-CNN [49], while historically important, was clearly outperformed, reflecting the limitations of purely convolutional designs. CTformer [18] and UNAD [50] recorded the weakest results, likely due to limited generalization under the severe noise levels present in this dataset. The superiority of the proposed method is further evident in Figure 4, where both full-slice- and ROI-based qualitative comparisons are presented. Reconstructions from De-TransGAN most closely resemble FDCT references, producing smoother noise profiles and sharper structural boundaries compared to other approaches. The visual evidence supports the quantitative findings, confirming that De-TransGAN achieves the most balanced trade-off between high PSNR, strong SSIM, and reduced RMSE. Overall, these results highlight the robustness of the proposed transformer-augmented GAN in handling previously unseen chest CT data, demonstrating its clinical promise under challenging low-dose conditions.

#### 7.1.2. Head CT Results

**Figure 6:**
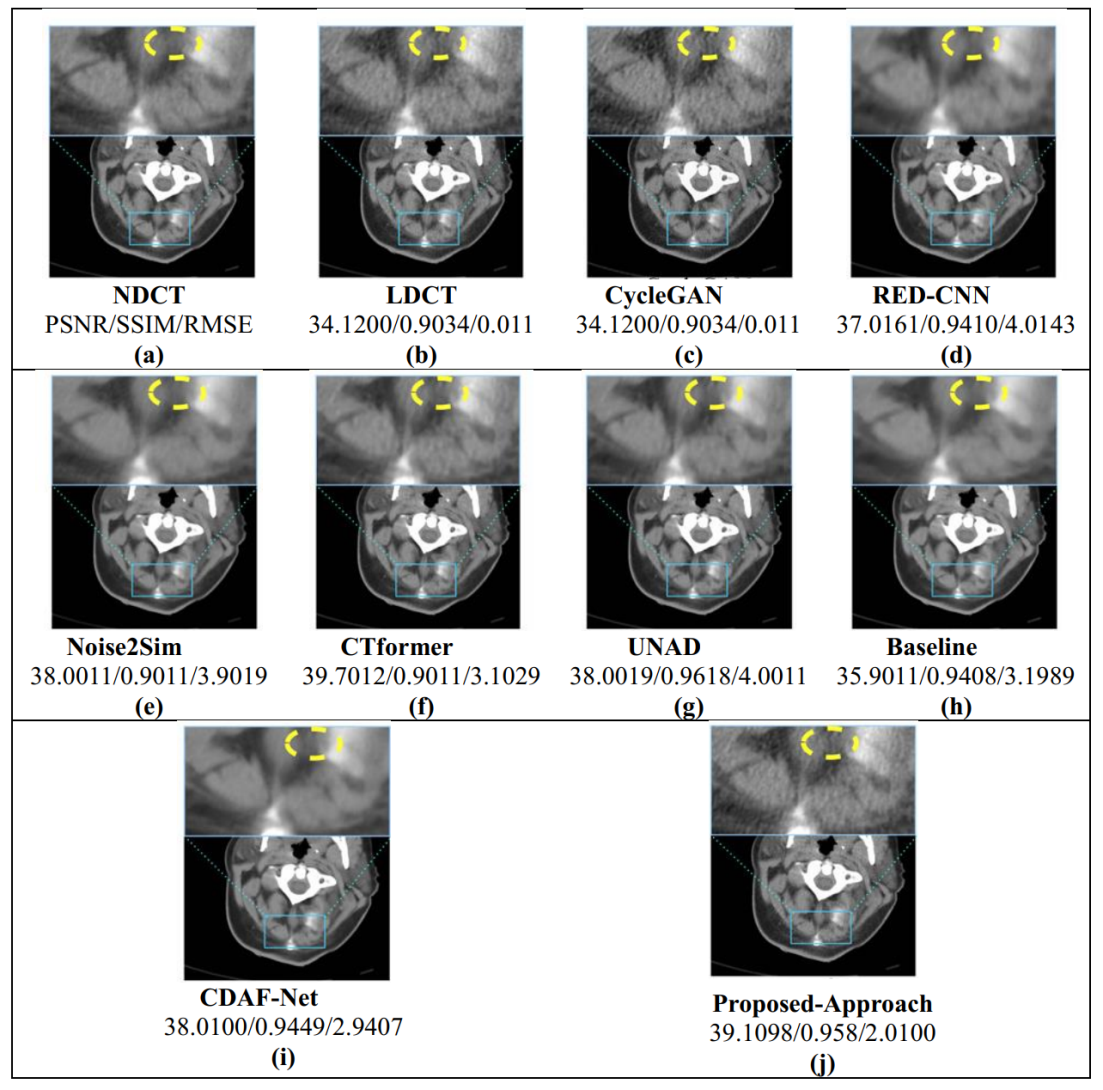
Simulation results of visual quality and perceptual effectiveness of the proposed approach with other denoising approaches based on unseen head CT images from the LDCT and Projection dataset.

**Figure 7:**
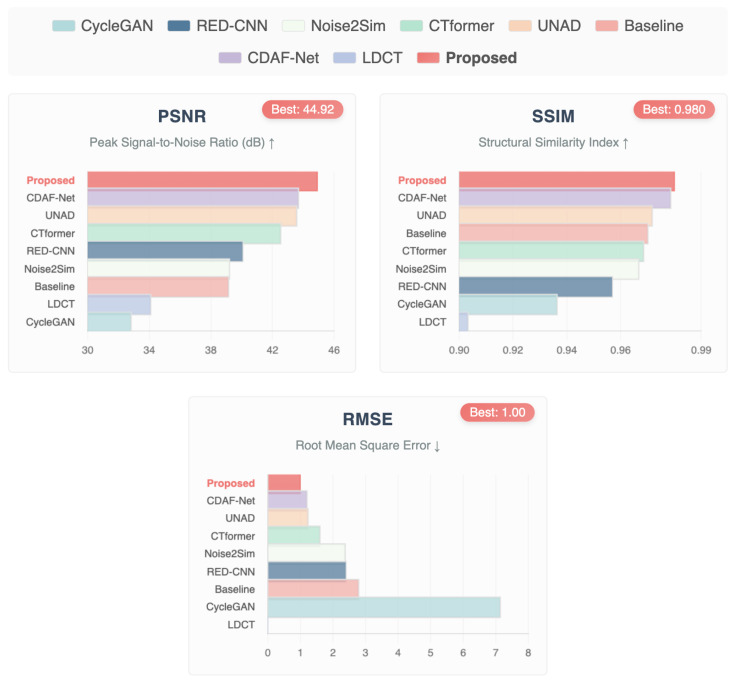
Comparative bar plots of PSNR, SSIM, and RMSE for head CT images from the LDCT and Projection dataset. The proposed De-TransGAN achieves the highest PSNR (44.92 dB) and SSIM (0.9801), along with the lowest RMSE (1.001), consistently outperforming state-of-the-art methods and demonstrating superior structural preservation and noise suppression. *Note: ↑ indicates higher values represent better performance; ↓ indicates lower values represent better performance*.

For the second evaluation, we investigated head CT images from the LDCT and Projection dataset to further assess the robustness of the proposed framework. The quantitative comparison in Table 3 provides a detailed benchmark against state-of-the-art methods. Among competing techniques, CDAF-Net [51] achieves strong results (PSNR: 43.71, SSIM: 0.9788, RMSE: 1.2126), indicating its ability to preserve fine structural details. UNAD [50] also performs competitively with slightly higher RMSE values, while CTformer [18], leveraging long-range dependency modeling, reports a PSNR of 42.56 and RMSE of 1.6087, demonstrating the benefits of transformer-based learning for structural recovery. RED-CNN [49], though historically important, shows lower SSIM and higher RMSE, reflecting the limitations of early CNN-based denoisers under low-dose settings. Similarly, CycleGAN [48] underperforms across all metrics, highlighting the challenges of cycle consistency alone in addressing high-noise CT reconstruction. Most importantly, the proposed De-TransGAN clearly surpasses all baselines, achieving the highest PSNR (44.92), the best SSIM (0.9801), and the lowest RMSE (1.001). These results highlight the superior ability of De-TransGAN to preserve both global image fidelity and local structural accuracy while effectively suppressing noise. The relatively small standard deviations across all metrics further confirm its robustness and consistency across different head CT cases. To complement these numerical findings, Figure 6 and the graphical plots in Figure 7 illustrate the comparative distribution of PSNR, SSIM, and RMSE. The proposed method consistently occupies the top position across all three indicators, with the bar plots providing a clear visual confirmation of its dominance over competing methods. Notably, the improvements in SSIM reflect enhanced structural preservation, while the lowest RMSE scores demonstrate reduced reconstruction error at a pixel level.

Qualitative inspection of the reconstructed head CT images in Figure 6 further supports these results. De-TransGAN delivers clearer, artifact-free reconstructions with sharper boundaries compared to other methods, many of which either retain residual noise or oversmooth delicate anatomical details. Collectively, these results establish De-TransGAN as a highly effective solution for LDCT head image denoising, striking an optimal balance between perceptual realism, structural fidelity, and diagnostic reliability.

### 7.2. Mayo Dataset Results

Table 4 summarizes the quantitative evaluation on the Mayo dataset, benchmarked with PSNR, SSIM, and RMSE. The results reveal a clear progression in performance from earlier CNN-based approaches to modern GAN- and transformer-driven methods. Among these, the proposed De-TransGAN consistently delivers the best outcomes, achieving a PSNR of 34.01 dB, an SSIM of 0.9330, and the lowest RMSE of 4.66. These results highlight the method’s ability to suppress complex noise patterns while preserving subtle anatomical structures such as soft-tissue boundaries and organ edges. The relatively small standard deviations across all three metrics further emphasize the reliability and robustness of the framework across different cases.

**Figure 8:**
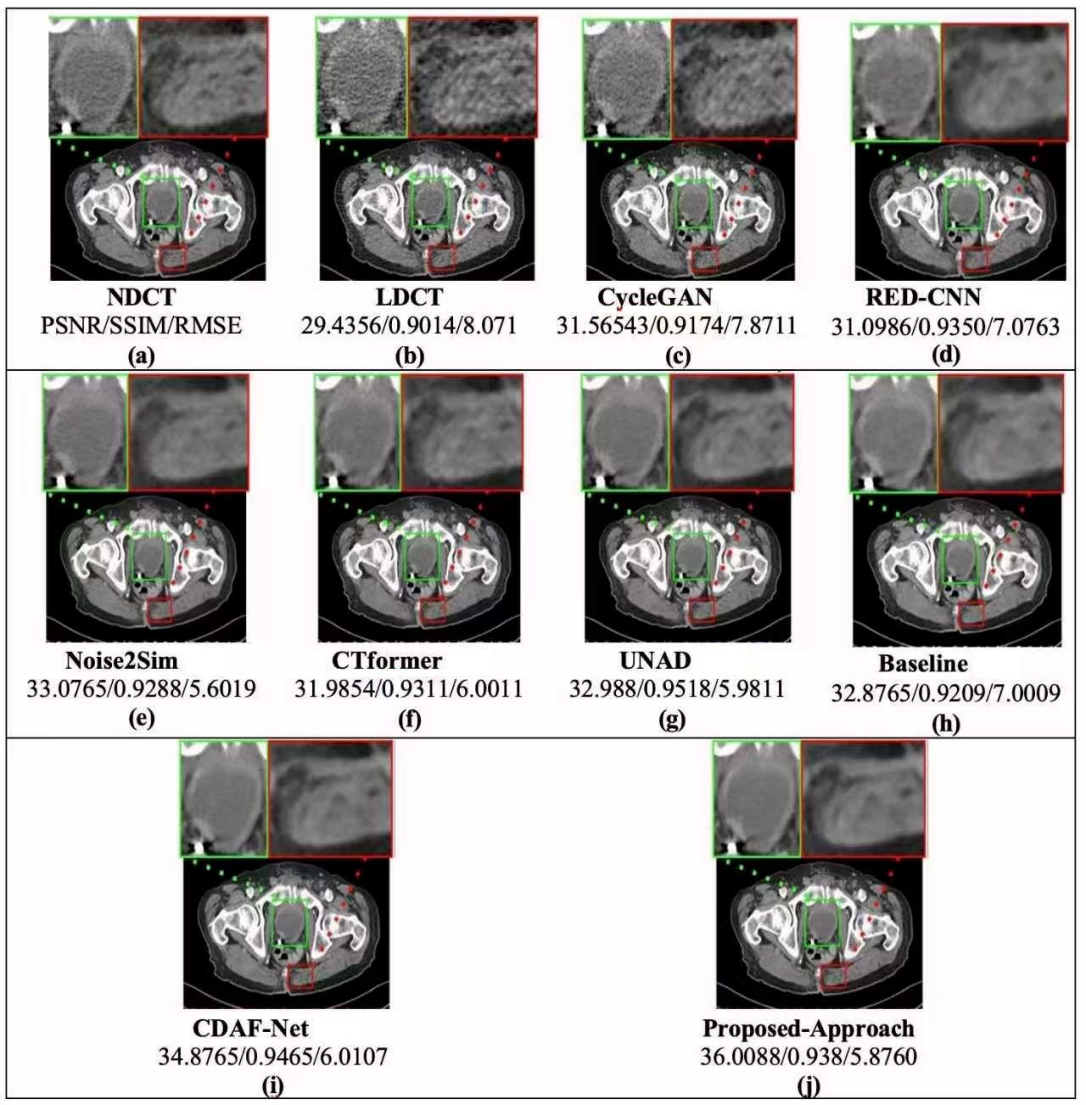
Results of visual quality and perceptual effectiveness of the proposed approach with other denoising approaches based on the Mayo dataset.

Figure 8 provides qualitative comparisons. While methods such as CycleGAN [48], RED-CNN [49], and CTformer [18] reduce some noise, they tend to leave residual streaks or blur finer anatomical detail. CDAF-Net [51] shows strong performance but still falls short of the structural fidelity achieved by De-TransGAN. In contrast, the proposed model produces reconstructions that are visually closest to FDCT references, faithfully recovering clinically important details such as bladder wall thickness and edge continuity.

**Figure 9:**
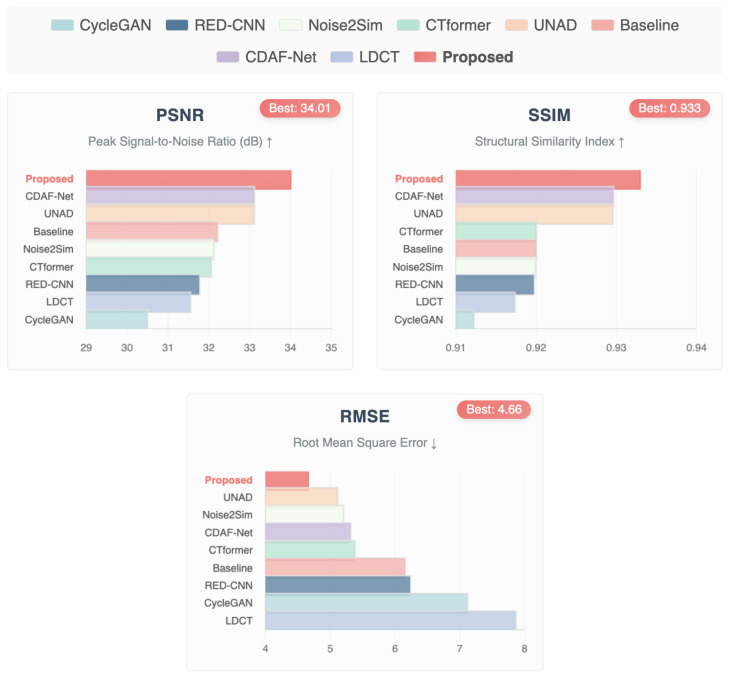
Comparative bar plots of PSNR, SSIM, and RMSE for Mayo CT images. The proposed De-TransGAN achieves the highest PSNR (34.01 dB) and SSIM (0.933), alongside the lowest RMSE (4.66), consistently outperforming state-of-the-art methods. *Note: ↑ indicates higher values represent better performance; ↓ indicates lower values represent better performance*.

Taken together, both the quantitative scores and graphical/visual analyses establish De-TransGAN as the most effective solution for Mayo data, achieving the optimal balance of noise suppression, structural preservation, and perceptual realism necessary for clinical deployment in low-dose CT imaging. The bar plots in Figure 9 (PSNR, SSIM, and RMSE) reinforce these findings. De-TransGAN demonstrates clear superiority over competing methods, with CDAF-Net [51] and UNAD [46] being the closest contenders but still falling short in at least one metric. Noise2Sim [45] and CTformer [18] achieve competitive PSNR and SSIM values, but their higher RMSE indicates instability in fine-detail recovery. RED-CNN [49] and CycleGAN [48], while reducing visible noise, perform substantially worse across all metrics, reflecting the limitations of earlier CNN- and cycle-consistency-driven designs.

### 7.3. Private Dataset

**Figure 10:**
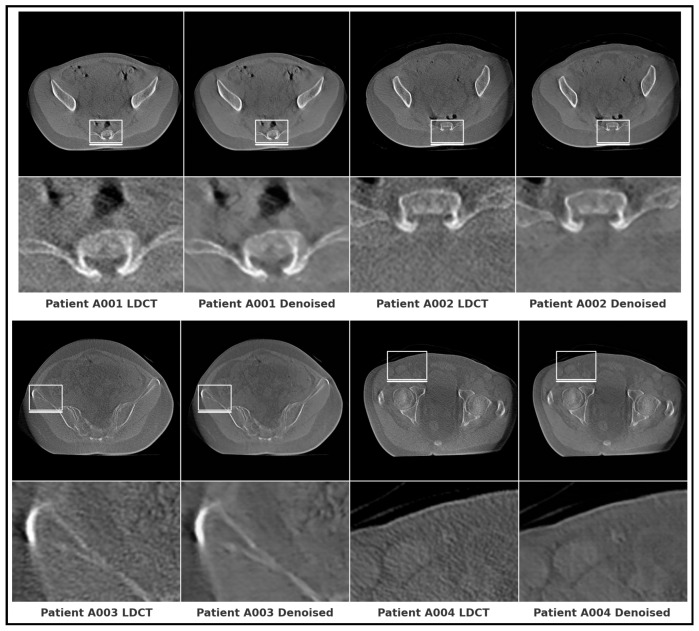
Representative qualitative results on the private low-dose CT dataset.

To further assess the real-world applicability and generalization capacity of the proposed framework, we conducted an independent evaluation on a private clinical dataset comprising four patients (A001–A004), each containing only low-dose CT (LDCT) scans. Unlike the public datasets used for supervised training and validation, this dataset was reserved exclusively for external qualitative testing, thereby providing an unbiased benchmark of robustness. As quantitative ground truth (FDCT) images were not available, evaluation was performed qualitatively. Visual inspection, shown in Figure 10, demonstrates that De-TransGAN effectively suppresses noise while preserving fine anatomical details such as tissue interfaces and bone edges. Across all four cases, the denoised images exhibit smoother noise profiles and clearer boundaries compared to the original LDCT inputs, confirming that the model maintains strong generalization under unseen clinical conditions.

## 8. Ablation Study and Analysis

To further validate the individual contribution of each core module within the proposed De-TransGAN framework, we conducted a comprehensive ablation study using the publicly available LDCT and Projection Data dataset. This study was designed to systematically analyze the effect of each architectural component on the overall denoising performance and image quality. All experiments were conducted under identical training configurations for a fixed number of fifty epochs to ensure fairness and isolate the impact of each removed element. For this analysis, we selected three representative patient cases from the test split, namely Patient C280, Patient C290, and Patient C295. These cases were chosen due to their variability in anatomical features and noise characteristics, providing a diverse evaluation benchmark. Both the input low-dose images and their corresponding denoised reconstructions were analyzed in detail, focusing on tissue boundaries, soft-tissue contrast, and the presence of residual artifacts.

Multiple model variants were constructed by selectively removing key modules from the full De-TransGAN architecture. The following configurations were evaluated: (i) a version without transformer blocks to assess the role of global context modeling, (ii) a version without the GAN adversarial learning component to assess its impact on perceptual sharpness, and (iii) a version without the CBAM attention mechanism to examine its contribution to regional feature emphasis and edge preservation.

**Figure 11:**
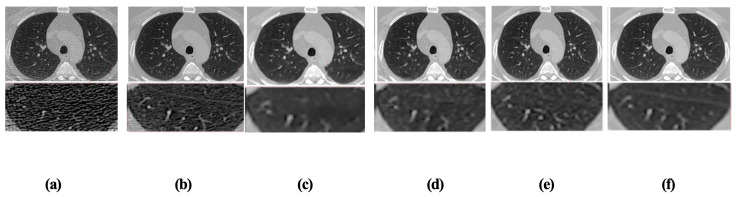
Simulation results of the ablation study on LDCT and Projection Data: (**a**) low-dose input; (**b**) full-dose reference; (**c**) De-TransGAN without transformer; (**d**) De-TransGAN without GAN component; (**e**) De-TransGAN without attention mechanism; and (**f**) full De-TransGAN with all components.

Qualitative comparisons of the resulting images from each variant are presented in Figure 11. The variant lacking transformer blocks, shown in Figure 11c, exhibited significant degradation in output quality. The absence of multi-head self-attention eliminated the model’s ability to learn long-range dependencies, resulting in blurry textures and indistinct organ boundaries. Subtle anatomical features, such as vascular edges and interlobar fissures, were often smoothed out or lost entirely. The version without the GAN discriminator, illustrated in Figure 11d, produced reconstructions that were structurally accurate in terms of intensity distribution but appeared overly smooth and lacked perceptual realism. The absence of adversarial learning led the generator to optimize purely pixel-level fidelity, resulting in images that were numerically sound but visually flat, with diminished fine textural contrast that is often critical in diagnostic interpretation. Similarly, when the CBAM attention mechanism was removed from the model, as shown in Figure 11e, the resulting images displayed inconsistent suppression of noise across different anatomical regions. Without attention-guided refinement, the network failed to prioritize diagnostically important areas such as tissue junctions, lesion borders, and airway contours. As a consequence, residual noise and irregular textures persisted, especially in low-contrast zones. In contrast, the complete De-TransGAN configuration, presented in Figure 11f, achieved the most consistent and diagnostically reliable results. The model preserved fine anatomical detail, delineated soft-tissue interfaces with clarity, and effectively suppressed background noise without introducing artifacts. Its performance closely resembled the full-dose ground truth images shown in Figure 11b, particularly in terms of structure continuity and edge sharpness. The detailed quantitative results for each ablation setting are summarized in Table 5.

This ablation study reinforces that each of the three components, transformer blocks, GAN adversarial loss, and CBAMs, plays a critical and complementary role in enhancing the model’s capability. The transformer blocks are essential for learning global semantic representations, the GAN component promotes perceptual realism by learning the manifold of natural CT textures, and the CBAMs provide fine-grained spatial- and channel-wise attention that ensures localized refinement. Their collective integration is indispensable for achieving the high-quality, high-fidelity denoising results that the full De-TransGAN model consistently demonstrates. These findings not only validate our architectural design but also highlight the necessity of each module in delivering robust and clinically applicable LDCT image reconstruction.

## 9. Conclusions and Future Work

This study introduced De-TransGAN, a transformer-augmented GAN framework for low-dose CT (LDCT) image denoising. By combining a TransGAN-based generator, a hybrid PatchGAN-ViT discriminator, CBAM attention, and a composite L1–SSIM–VGG loss, the model effectively balances global context, local structural fidelity, and perceptual realism. Evaluations on the TCIA LDCT and Projection dataset, AAPM Mayo Clinic, and a private clinical dataset demonstrated state-of-the-art performance. On the Mayo dataset, De-TransGAN achieved a PSNR of 34.01 dB, SSIM of 0.9330, and RMSE of 4.66, outperforming leading methods such as RED-CNN, CycleGAN, and CDAF-Net. Results on the private dataset further confirmed its generalization ability, producing reconstructions with high anatomical fidelity. Future work will focus on two key directions: (1) zero-shot denoising with unpaired data, training only on FDCT images to eliminate the need for hardly available paired datasets, and (2) direct image reconstruction from projection data, bypassing LDCT/FDCT intermediates. Additional efforts will target lightweight transformers, domain generalization, and explainable AI to ensure scalable, trustworthy deployment. In conclusion, De-TransGAN sets a strong foundation for next-generation LDCT imaging, offering both state-of-the-art denoising performance and a clear roadmap toward zero-shot learning and projection-driven reconstruction. 

## Figures and Tables

**Table 1:** Critical analysis of the different denoising approaches. *Note: ↑ indicates higher values represent better performance; ↓ indicates lower values represent better performance*.

Article Title	Problem Addressed	Major Contributions	Datasets Used	Development Environment	Quantitative Results	Limitations
CGP–Uformer (2023)	Noise and detail loss in LDCT	Channel Graph Perception in Uformer for improved denoising	AAPM Mayo Clinic 2016	PyTorch, NVIDIA 3090 Ti, LR = 2 × 10^−4^	PSNR: 35.56 ± 3.69 dB, SSIM: 0.9221	Dataset-specific tuning; large memory footprint
Multi-Attention U-Net (2023)	Ineffective feature extraction in noisy CT	Multi-level attention (local, channel, hierarchical) for better detail preservation	QIN_LUNG_CT, Mayo Clinic	Not Specified	PSNR: 34.73/28.91 dB, SSIM: 0.93/0.86	No transformer component; limited generalization
CFAN-Net (2023)	Structure loss and blurry recon in LDCT	Compound attention + edge enhancement for feature recovery	Mayo LDCT (35 images)	Not Specified	PSNR: 33.97 ± 0.40, SSIM: 0.9198	Limited dataset size; lacks real-time feasibility
Hformer (2023)	Lack of global context in CNN-based denoisers	Vision transformer with residual learning and scale encoding	Not mentioned	Not Specified	Qualitative only; no exact PSNR/SSIM	Results not numerically validated
SIST (2022)	Noise in sinogram domain before image reconstruction	Sinogram-based transformer model	LDCT, simulated datasets	Not Specified	PSNR: up to 44.74 dB, SSIM: 0.916	Requires access to sinogram data; complex pipeline
Transformer-based GAN (Zhang et al., 2024)	Sparse-view artifacts in CT	Attention-based GAN for artifact removal	NIH-AAPM 2016	Not Specified	Qualitatively better than CTTR, PRestor	Sparse-view focus; not evaluated on full-dose denoising
Eformer (2021)	Edge blurring in denoised CT images	Transformer + learnable Sobel filters for edge enhancement	AAPM Mayo Clinic	PyTorch	PSNR: 43.487, SSIM: 0.9861	Early design; no attention to runtime efficiency
Deep RL Denoiser (2022)	Parameter-heavy deep models	Low-param deep RL with bilateral filtering	AAPM Mayo Clinic, TCIA	PyTorch + Deep RL	PSNR ↑ by 0.4 dB, SSIM ↑ by 0.025	Marginal improvement; tuning complexity
GAN Post-Processing (Zhang et al., 2024)	Noise in reconstructed CT	Post-processing GAN with detail restoration module	Not specified	Not Specified	Reported qualitative improvement	No clear numerical benchmarks
Veterinary GAN (You et al., 2024)	Noise in LDCT for canines	GAN with anti-aliasing generator and multiscale discriminator	Veterinary LDCT images	Not Specified	Improved image clarity (qualitative)	Application-specific; small dataset
Narrative Review (Chen et al., 2022)	Lack of comprehensive synthesis of GAN+attention	Review of attention-based GANs in medical imaging	Multiple	Literature Review	NA	Not experimental
CTformer (2022)	Convolutional limitations in LDCT denoising	Token2Token Vision Transformer (no CNNs)	Mayo LDCT	Not Specified	Higher PSNR and SSIM vs. RED-CNN, DnCNN	May be sensitive to token size and stride

**Table 2:** Comparative study and analysis of various denoising approaches on chest CT images from the LDCT and Projection dataset. *Note: ↑ indicates higher values represent better performance; ↓ indicates lower values represent better performance*.

Method	PSNR (a ± b) ↑	SSIM (a ± b) ↑	RMSE (a ± b) ↓
CycleGAN [52]	19.3456 ± 1.5587	0.7487 ± 0.0507	26.7243 ± 4.7021
RED-CNN [49]	16.1256 ± 2.1179	0.7598 ± 0.0571	38.0832 ± 8.9821
Noise2Sim [50]	20.1322 ± 2.0211	0.7679 ± 0.0546	26.1825 ± 6.1034
CTformer [18]	15.1290 ± 2.0199	0.7289 ± 0.0584	44.7713 ± 8.1150
UNAD [53]	16.1034 ± 2.3375	0.7639 ± 0.0602	41.8112 ± 8.4163
Baseline [47]	19.9810 ± 1.9332	0.7588 ± 0.0490	26.4719 ± 5.6704
CDAF-Net [51]	20.1398 ± 1.9290	0.7758 ± 0.0504	24.3264 ± 5.1858
**Proposed Approach **	**21.2631 ± 0.3498**	**0.7816 ± 0.0256**	**23.1265 ± 5.0100**

**Table 3:** Comparative study and analysis of various denoising approaches on head CT images from the LDCT and Projection dataset. *Note: ↑ indicates higher values represent better performance; ↓ indicates lower values represent better performance*.

Method	PSNR (a ± b) ↑	SSIM (a ± b) ↑	RMSE (a ± b) ↓
CycleGAN [52]	32.8390 ± 3.1109	0.9365 ± 0.1269	7.1480 ± 4.5423
RED-CNN [49]	40.1007 ± 2.0333	0.9571 ± 0.2112	2.4135 ± 0.3068
Noise2Sim [50]	39.2310 ± 2.7016	0.9669 ± 0.3103	2.3839 ± 0.4921
CTformer [18]	42.5591 ± 2.5753	0.9687 ± 0.2112	1.6087 ± 0.3658
UNAD [53]	43.5916 ± 2.5373	0.9719 ± 0.0108	1.2424 ± 0.7623
Baseline [47]	39.1719 ± 1.9501	0.9703 ± 0.0292	2.7984 ± 0.3526
CDAF-Net [51]	43.7136 ± 2.4209	0.9788 ± 0.0392	1.2126 ± 0.6220
**Proposed Approach**	**44.9217 ± 2.2391**	**0.9801 ± 0.0362**	**1.001 ± 0.1021**

**Table 4:** Comparative study and analysis of various denoising approaches on the Mayo dataset. *Note: ↑ indicates higher values represent better performance; ↓ indicates lower values represent better performance*.

Method	PSNR (a ± b) ↑	SSIM (a ± b) ↑	RMSE (a ± b) ↓
CycleGAN [52]	30.5123 ± 1.3212	0.9123 ± 0.0322	7.1261 ± 1.9012
RED-CNN [49]	31.7823 ± 1.7500	0.9198 ± 0.0214	6.2430 ± 1.3275
Noise2Sim [50]	32.1284 ± 1.1430	0.9200 ± 0.0143	5.2130 ± 1.2354
CTformer [18]	32.0708 ± 1.2762	0.9201 ± 0.0342	5.3891 ± 1.4160
UNAD [53]	33.1256 ± 1.1875	0.9296 ± 0.0233	5.1201 ± 1.3520
Baseline [47]	32.2311 ± 1.1208	0.9201 ± 0.0167	6.1630 ± 1.1542
CDAF-Net [51]	33.1276 ± 1.2897	0.9297 ± 0.0364	5.3231 ± 1.0036
**Proposed Approach**	**34.0111 ± 1.6753**	**0.9330 ± 0.0212**	**4.6633 ± 1.6534**

**Table 5:** Quantitative ablation study on the LDCT and Projection Data dataset (Patients C280, C290, C295). *Note: ↑ indicates higher values represent better performance; ↓ indicates lower values represent better performance*.

Model Variant	PSNR (dB) ↑	SSIM ↑	RMSE ↓
Full De-TransGAN (Proposed)	21.2631	0.7816	23.1265
Without Transformer Blocks	20.5112	0.7502	25.3953
Without GAN Adversarial Component	20.1349	0.7394	26.1428
Without CBAM Attention	20.3928	0.7447	25.6017

## Data Availability

The LDCT and Projection Data collection is publicly available at The Cancer Imaging Archive (TCIA) under the title “Low Dose CT Image and Projection Data (LDCT and Projection data)” (https://doi.org/10.7937/9NPB-2637 (accessed on 27 February 2025)). The AAPM Mayo Clinic Low Dose CT Grand Challenge dataset is also publicly accessible through the American Association of Physicists in Medicine (AAPM) website (https://www.aapm.org/GrandChallenge/LowDoseCT/ (accessed on 27 February 2025)). The private clinical dataset used in this study was provided by a collaborating hospital under ethical and confidentiality agreements and is not publicly available due to patient privacy restrictions.

## Abbreviations

The following abbreviations are used in this manuscript:

CBAM	Convolutional Block Attention Module
CNN	Convolutional Neural Network
GAN	Generative Adversarial Network
LDCT	Low-Dose Computed Tomography
FDCT	Full-Dose Computed Tomography
MRI	Magnetic Resonance Imaging
MSE	Mean Squared Error
PSNR	Peak Signal-to-Noise Ratio
SSIM	Structural Similarity Index Measure
De-TransGAN	Denoising Transformer-Augmented Generative Adversarial Network
ViT	Vision Transformer
WGAN-GP	Wasserstein GAN with Gradient Penalty
